# DNA Binding Compatibility of the *Streptococcus pneumoniae* SsbA and SsbB Proteins

**DOI:** 10.1371/journal.pone.0024305

**Published:** 2011-09-07

**Authors:** Brenda Salerno, Geetha Anne, Floyd R. Bryant

**Affiliations:** Department of Biochemistry and Molecular Biology, Bloomberg School of Public Health, Johns Hopkins University, Baltimore, Maryland, United States of America; University of South Florida College of Medicine, United States of America

## Abstract

**Background:**

*Streptococcus pneumoniae* has two paralogous, homotetrameric, single-stranded DNA binding (SSB) proteins, designated SsbA and SsbB. Previous studies demonstrated that SsbA and SsbB have different solution-dependent binding mode preferences with variable DNA binding capacities. The impact of these different binding properties on the assembly of multiple SsbAs and SsbBs onto single-stranded DNA was investigated.

**Methodology/Principal Findings:**

The complexes that were formed by the SsbA and SsbB proteins on dT*_n_* oligomers of defined lengths were examined by polyacrylamide gel electrophoresis. Complexes containing either two SsbAs or two SsbBs, or mixed complexes containing one SsbA and one SsbB, could be formed readily, provided the dT*_n_* oligomer was long enough to satisfy the full binding mode capacities of each of the bound proteins under the particular solution conditions. Complexes containing two SsbAs or two SsbBs could also be formed on shorter dT*_n_* oligomers via a “shared-strand binding” mechanism in which one or both proteins were bound using only a portion of their potential binding capacity. Mixed complexes were not formed on these shorter oligomers, however, indicating that SsbA and SsbB were incompatible for shared-strand binding. Additional experiments suggested that this shared-strand binding incompatibility may be due in part to differences in the structure of a loop region on the outer surface of the subunits of the SsbA and SsbB proteins.

**Conclusion/Significance:**

These results indicate that the SsbA and SsbB proteins may co-assemble on longer DNA segments where independent binding is possible, but not on shorter DNA segments where coordinated interactions between adjacent SSBs are required. The apparent compatibility requirement for shared-strand binding could conceivably serve as a self-recognition mechanism that regulates the manner in which SsbA and SsbB interact in *S. pneumoniae*.

## Introduction

**Figure 1:**
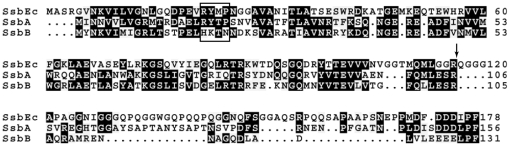
*Streptococcus pneumoniae* SsbA and SsbB proteins. The amino acid sequences of the *S. pneumoniae* SsbA and SsbB proteins are aligned with that of the *E. coli* SSB protein, SsbEc. Identical residues are highlighted in black. The division between the N-terminal and C-terminal domains is indicated by an arrow, and the putative Loop 1 region is denoted with a box.

The naturally transformable Gram-positive bacterium *Streptococcus pneumoniae* has two paralogous, homotetrameric, single-stranded DNA binding (SSB) proteins, designated SsbA and SsbB (Figure 1) [1]–[3]. The SsbA protein (156 amino acids/17,350 Da per monomer) is expressed constitutively whereas the SsbB protein (131 amino acids/14,926 Da per monomer) is induced specifically during transformational competence. These expression patterns suggest that the SsbA protein may serve as a general SSB protein for routine DNA functions, and that the SsbB protein may be a specialized SSB protein used primarily during natural transformation [1].

The N-terminal domains of the SsbA and SsbB proteins (amino acids 1–105/106) are similar in sequence. The C-terminal domain of the SsbB protein (amino acids 106–131), however, is significantly shorter than that of the SsbA protein (amino acids 107–156) (Figure 1). Structural studies of the corresponding regions of the homotetrameric SSB protein from *Escherichia coli* (SsbEc) and other bacterial SSB proteins have shown that the N-terminal domain contains the DNA binding and subunit tetramerization sites, whereas the C-terminal domain may serve as a binding site for other proteins involved in various DNA functions [4].

Bacterial SSB proteins bind single-stranded DNA in a non-sequence-specific manner. The DNA binding properties of the SsbEc protein have been the most extensively characterized. Two major binding modes have been identified: the SSB_35_ mode and the SSB_65_ mode. In the SSB_35_ mode (favored at lower salt concentrations), two subunits of the SsbEc tetramer interact with the single-stranded DNA (occluding ∼35 nucleotides per tetramer), whereas in the SSB_65_ mode (favored at higher salt concentrations), all four subunits of the SsbEc tetramer interact with the single-stranded DNA (occluding ∼65 nucleotides per tetramer) [5].

We previously carried out a comparative analysis of the DNA binding mode properties of the SsbEc, SsbA, and SsbB proteins. In that study, the various SSB proteins were incubated with the oligomer, dT_35_, under different solution conditions and the resulting complexes were examined by polyacrylamide gel electrophoresis. In standard reaction solution (25 mM Tris acetate (pH 7.5)), the SsbEc protein was able to bind a single dT_35_ molecule, consistent with the SSB_35_ mode of binding. When Mg^2+^ (10 mM) was included in the reaction solution, however, the SsbEc protein was able to bind two dT_35_ molecules, consistent with the SSB_65_ mode of binding. The SsbA protein behaved similarly to the SsbEc protein under all reaction conditions, indicating that it interacted with dT_35_ in SSB_35_ and SSB_65_ modes that were analogous to those of the SsbEc protein. The SsbB protein, in contrast, appeared to bind two dT_35_ molecules in an SSB_65_-like mode in the absence of Mg^2+^, and in an enhanced SSB_65_-like mode (with positive intersubunit cooperativity) in the presence of Mg^2+^ [2].

The pronounced difference in binding mode preferences raises the question of whether SsbA and SsbB would be able to interact together on single-stranded DNA. To address this issue, we have now examined the assembly of multiple SsbAs or SsbBs on dT*_n_* oligomers of various defined lengths. Polyacrylamide gel electrophoresis was particularly well suited for this analysis because the various SSB·dT*_n_* complexes were readily resolvable and remarkably stable during electrophoresis, and the effect of solution conditions on complex formation could be assessed by varying the composition of the electrophoresis running buffer. The results indicate that: i) different mechanisms of assembly are available to the SsbA and SsbB proteins, depending on the length of the DNA and the specific solution conditions, and ii) SsbA and SsbB may co-assemble on longer DNA segments where independent binding is possible, but not on shorter DNA segments where coordinated interactions between adjacent SSBs are required.

## Results

### Experimental Design

The binding of the *Streptococcus pneumoniae* SsbA and SsbB proteins to a set of dT*_n_* oligomers ranging in length from dT_50_ to dT_130_ was examined. Particular attention was placed on determining the shortest dT*_n_* oligomer that was able to accommodate the binding of two SsbAs, two SsbBs, or one SsbA and one SsbB, in either the absence or presence of Mg^2+^. The expectation with this approach was that two SSBs would have to interact in a coordinated manner to form a complex on a minimal length dT*_n_*, whereas the SSBs would be able to bind independently to isolated sites on longer dT*_n_* oligomers. All binding reactions were carried out in solutions containing 25 mM Tris acetate (pH 7.5) and either 0 or 10 mM magnesium acetate, and the resulting complexes were analyzed by polyacrylamide gel electrophoresis using a running buffer identical in composition to that of the individual reaction solutions.

### SsbA protein assembly

**Figure 2:**
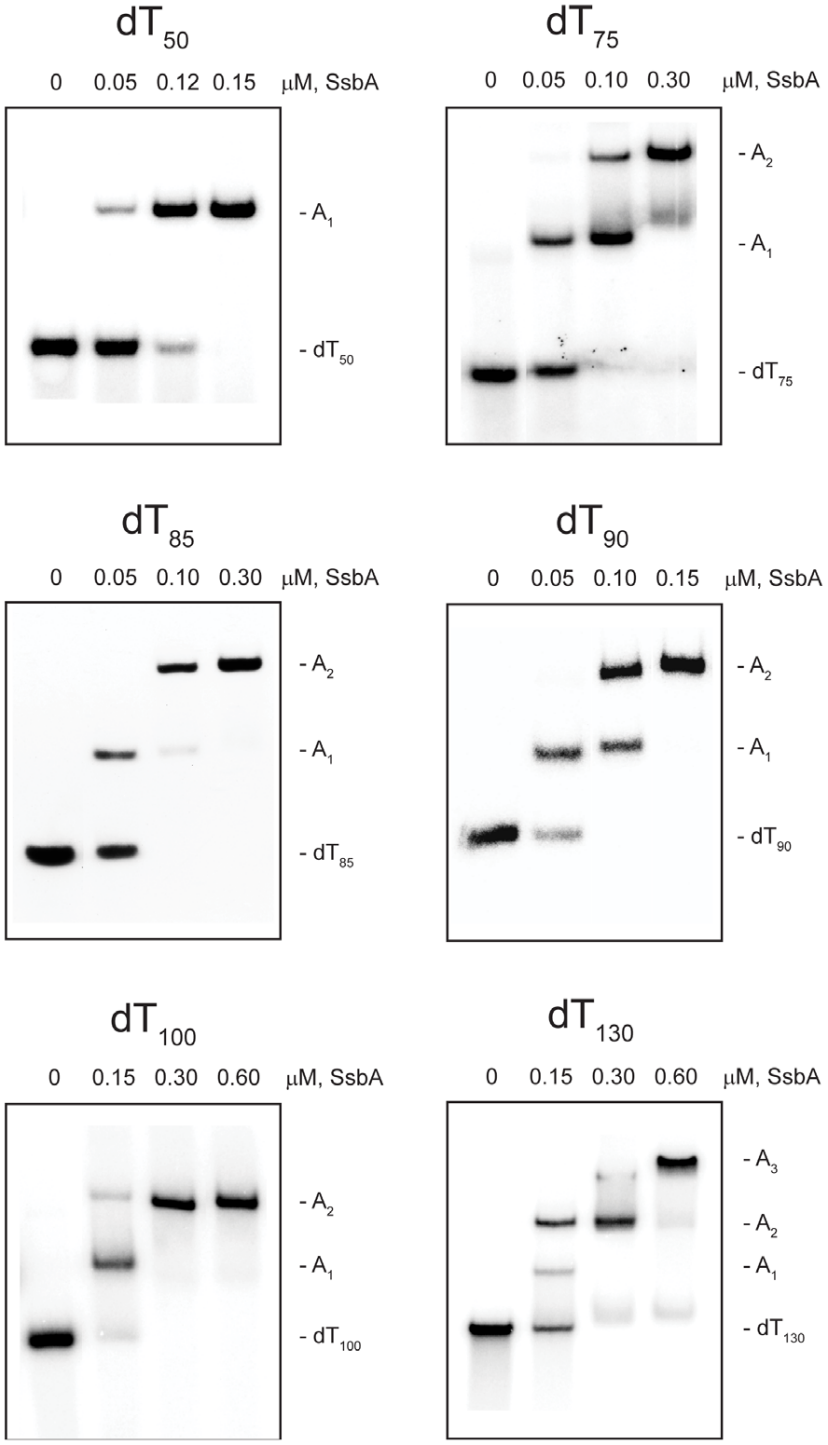
Binding of SsbA protein to dT*_n_* oligomers in the absence of Mg^2+^. The reaction solutions contained 25 mM Tris acetate (pH 7.5), 5 µM dT*_n_* (nucleotide concentration), and the indicated concentrations of SsbA protein (tetramer concentrations). The reactions were analyzed by polyacrylamide gel electrophoresis using a gel running buffer consisting of Tris acetate (pH 7.5). The bands corresponding to the unbound dT*_n_* oligomers, and the A_1_, A_2_, and A_3_ complexes, were visualized by phosphorimaging.

**Figure 3 pone-0024305-g003:**
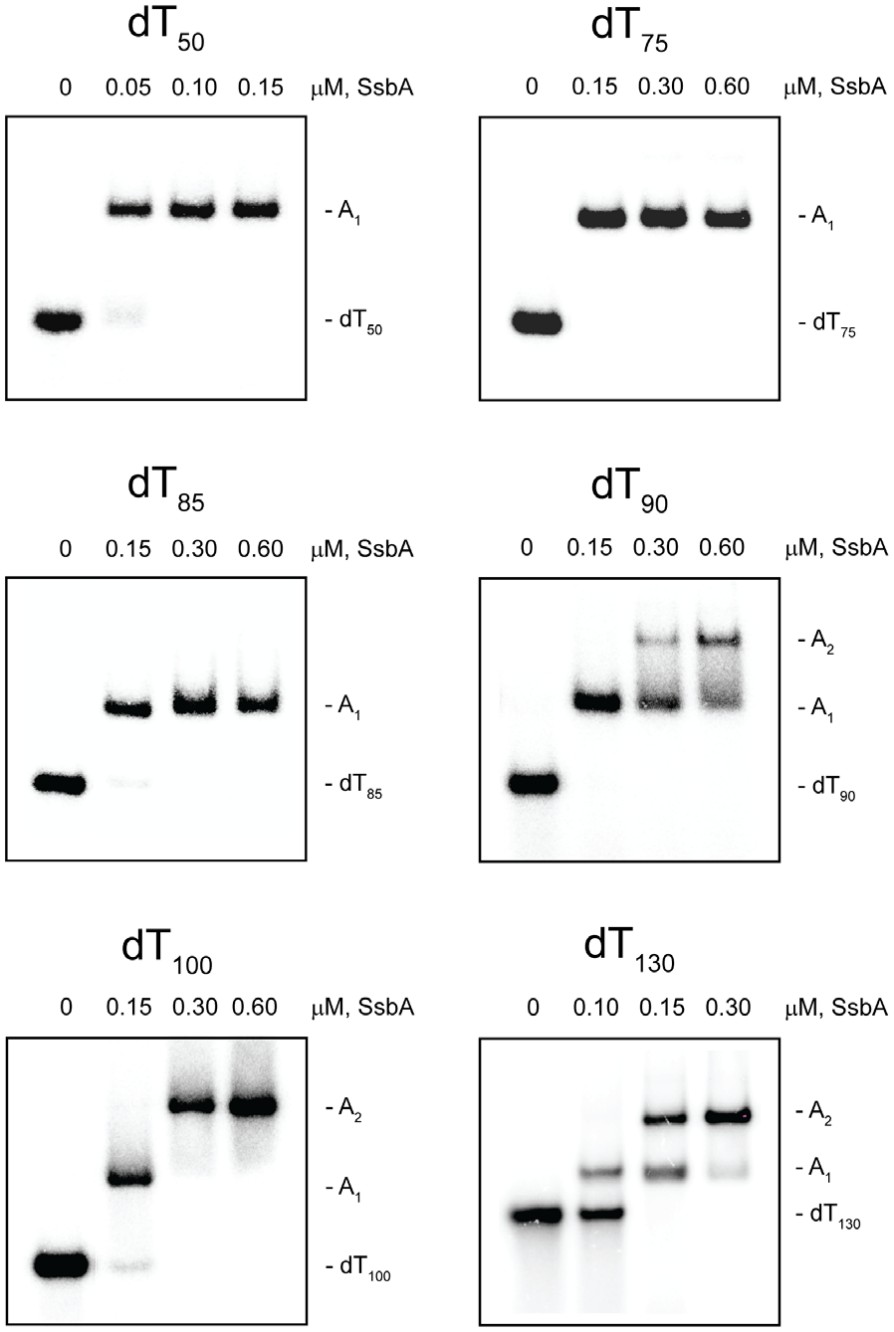
Binding of SsbA protein to dT*_n_* oligomers in the presence of Mg^2+^. The reaction solutions contained 25 mM Tris acetate (pH 7.5), 10 mM magnesium acetate, 5 µM dT*_n_* (nucleotide concentration), and the indicated concentrations of SsbA protein (tetramer concentrations). The reactions were analyzed by polyacrylamide gel electrophoresis using a gel running buffer consisting of Tris acetate (pH 7.5) and 10 mM magnesium acetate. The bands corresponding to the unbound dT*_n_* oligomers, and the A_1_ and A_2_ complexes, were visualized by phosphorimaging.

The complexes that were formed by the SsbA protein with the various dT*_n_* oligomers in the absence and presence of Mg^2+^ are shown in Figures 2 and 3, respectively (*note*: in these experiments, the electrophoretic mobilities of the free dT*_n_* oligomers exhibit a greater inverse-dependence on length than do the corresponding SsbA·dT*_n_* complexes, leading to a progressive decrease in the separation between the free dT*_n_* oligomers and the SsbA·dT*_n_* complexes with increasing dT*_n_* length).

#### Absence of Mg^2+^

When increasing concentrations of SsbA were added to dT_50_ in the absence of Mg^2+^, a single complex with a gel mobility lower than that of the unbound dT_50_ was formed (A_1_ complex). All of the dT_50_ was converted to this complex at an SsbA concentration that corresponded to approximately one SsbA tetramer per dT_50_ molecule, and there was no indication of the formation of a second complex at higher SsbA concentrations (Figure 2 and additional data not shown). A similar pattern of binding was obtained with the longer oligomer, dT_65_ (gel not shown). These results indicated that a single SsbA was able to bind to dT_50_ and dT_65_ under these reaction conditions.

When the oligomer length was increased to dT_75_, an A_1_ complex was formed with increasing SsbA concentrations in a manner similar to that observed with the shorter oligomers. As the concentration of SsbA was increased further, however, the A_1_ complex disappeared and a new complex with an even lower gel mobility was formed (A_2_ complex). This result indicated that a second SsbA was able to bind to dT_75_ under these conditions. A similar pattern of binding was observed with the longer oligomers, dT_85_, dT_90_, and dT_100_, indicating that two SsbAs were able to bind to each of these oligomers as well (Figure 2).

When the oligomer length was increased to dT_130_, A_1_ and A_2_ complexes were formed with increasing SsbA concentrations in a manner similar to that observed with dT_100_. At higher SsbA concentrations, however, a third complex with a mobility lower than either the A_1_ and A_2_ complexes was formed (A_3_ complex). This result indicated that three SsbAs were able to bind to dT_130_ under these conditions (Figure 2).

The results in Figure 2 indicated that the shortest dT*_n_* oligomer in the set that was able to bind two SsbAs in the absence of Mg^2+^ was dT_75_, and the shortest dT*_n_* that was able to bind three SsbAs was dT_130_. These results are summarized in Table 1.

**Table 1:** Complexes formed by the SsbA and SsbB proteins on dT*_n_* oligomers.

	SsbA		SsbB	
dT*_n_*	−Mg^2+^	+Mg^2+^	−Mg^2+^	−Mg^2+^
dT_50_	A_1_	A_1_	B_1_	B_1_
dT_65_	A_1_	A_1_	B_1_	B_1_
dT_75_	A_2_	A_1_	B_1_	B_1_
dT_85_	A_2_	A_1_	B_2_	B_1_
dT_90_	A_2_	A_2_	B_2_	B_1_
dT_100_	A_2_	A_2_	B_2_	B_2_
dT_130_	A_3_	A_2_	B_2_	B_2_

These results were derived from the experiments shown in Figures 2–5 and additional experiments (gels not shown), and indicate the highest order complexes that were observed when the indicated dT*_n_* oligomers were mixed with an excess concentration of SsbA or SsbB protein, in the absence or presence of 10 mM Mg^2+^ (the notations A*_n_* and B*_n_* indicate the number of SsbAs or SsbBs bound).

#### Presence of Mg^2+^

When increasing concentrations of SsbA were added to dT_50_ in the presence of Mg^2+^, a single complex with a gel mobility lower than that of the unbound dT_50_ was formed (A_1_ complex), with no indication of the formation of a second complex at higher SsbA concentrations (Figure 3). A similar pattern of binding was obtained with dT_65_ (gel not shown). These results were similar to those that were obtained in the absence of Mg^2+^ and indicated that a single SsbA was able to bind to dT_50_ and dT_65_ in the presence of Mg^2+^. A_1_ complexes were also formed when increasing concentrations of SsbA were added to the longer oligomers, dT_75_ and dT_85_, but in contrast to the results that were obtained in the absence of Mg^2+^, A_2_ complexes were not detected with these oligomers (Figure 3).

When the oligomer length was increased to dT_90_, an A_1_ complex was formed at lower SsbA concentrations in a manner similar to that observed with the shorter oligomers. As the concentration of SsbA was increased further, however, the A_1_ complex disappeared and a new complex with an even lower gel mobility was formed (A_2_ complex). This result indicated that a second SsbA was able to bind to dT_90_ under these conditions. A similar pattern of binding was obtained with dT_100_ and dT_130_, indicating that two SsbAs were able to bind to each of these oligomers as well. In contrast to the results that were obtained in the absence of Mg^2+^, however, there was no indication of the formation of a third complex with dT_130_, even at the highest concentrations of SsbA that were examined (Figure 3).

The results in Figure 3 indicated that the shortest dT*_n_* oligomer in the set that was able to bind two SsbAs in the presence of Mg^2+^ was dT_90_, and that only two SsbAs were able to bind even when the oligomer length was increased to dT_130_. These results are summarized in Table 1.

### SsbB protein assembly

**Figure 4 pone-0024305-g004:**
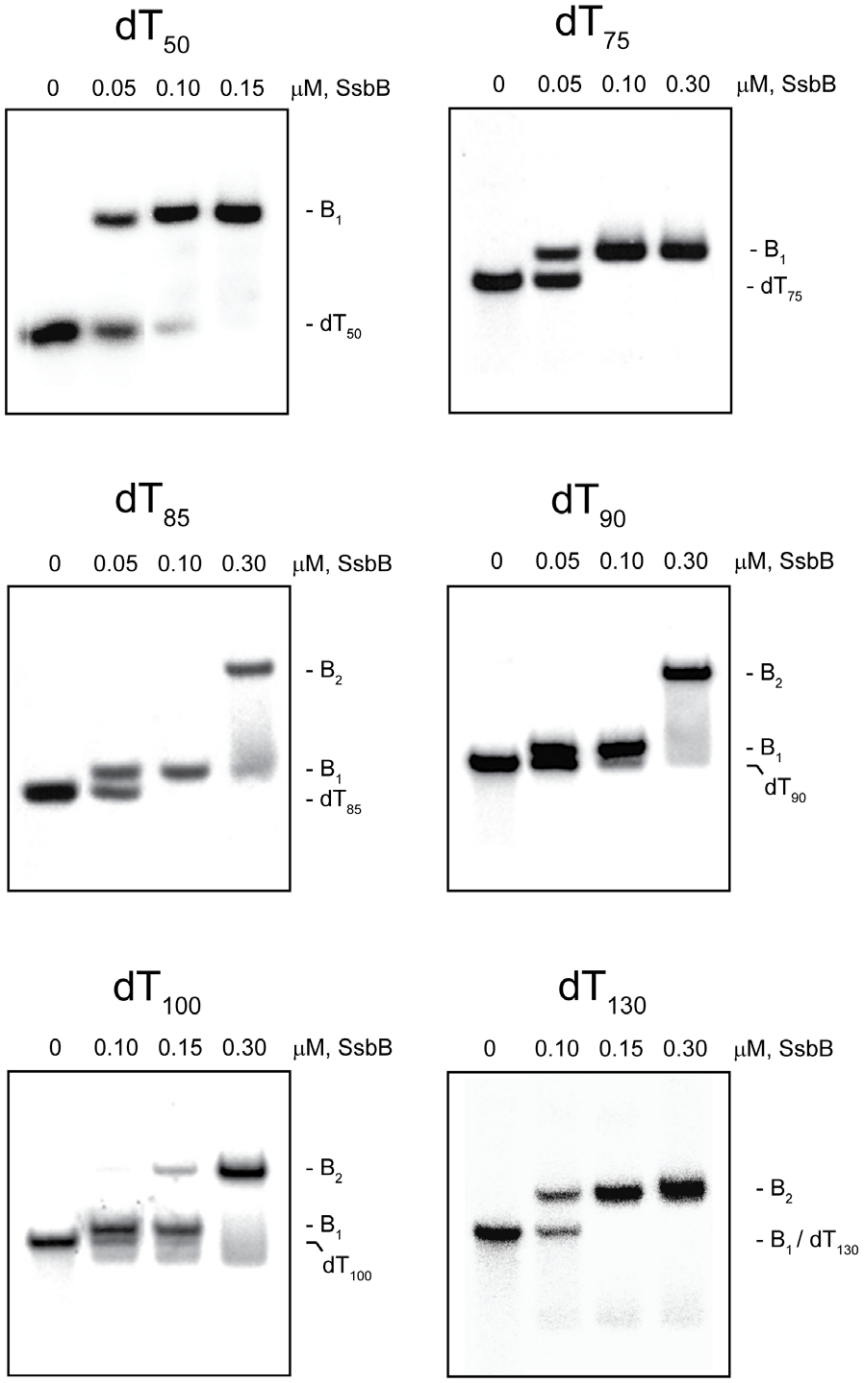
Binding of SsbB protein to dT*_n_* oligomers in the absence of Mg^2+^. The reaction solutions contained 25 mM Tris acetate (pH 7.5), 5 µM dT*_n_* (nucleotide concentration), and the indicated concentrations of SsbB protein (tetramer concentrations). The reactions were analyzed by polyacrylamide gel electrophoresis using a gel running buffer consisting of Tris acetate (pH 7.5). The bands corresponding to the unbound dT*_n_* oligomers, and the B_1_ and B_2_ complexes, were visualized by phosphorimaging.

**Figure 5 pone-0024305-g005:**
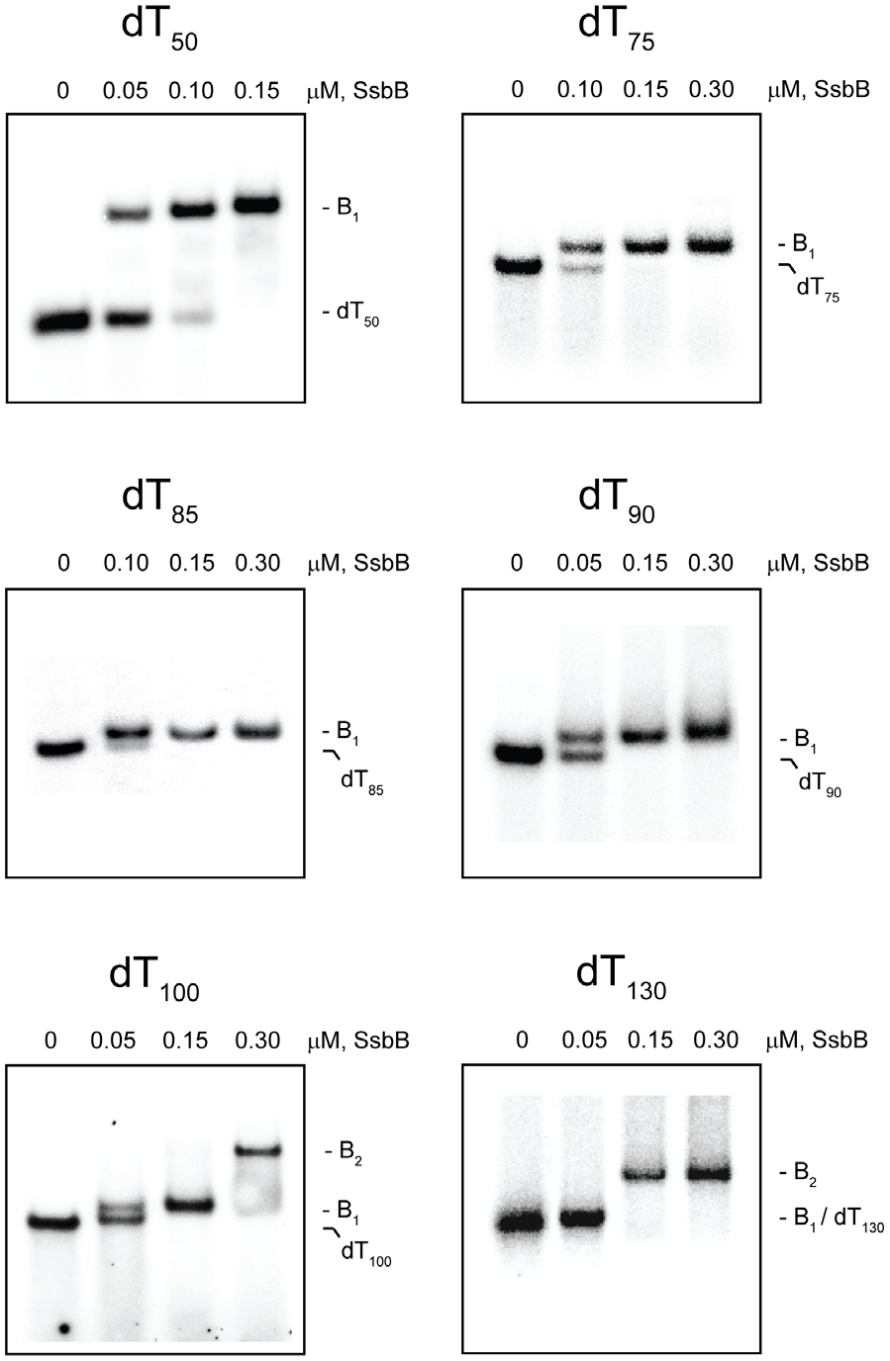
Binding of SsbB protein to dT*_n_* oligomers in the presence of Mg^2+^. The reaction solutions contained 25 mM Tris acetate (pH 7.5), 10 mM magnesium acetate, 5 µM dT*_n_* (nucleotide concentration), and the indicated concentrations of SsbB protein (tetramer concentrations). The reactions were analyzed by polyacrylamide gel electrophoresis using a gel running buffer consisting of Tris acetate (pH 7.5) and 10 mM magnesium acetate. The bands corresponding to the unbound dT*_n_* oligomers, and the B_1_ and B_2_ complexes, were visualized by phosphorimaging.

The complexes that were formed by the SsbB protein with the various dT*_n_* oligomers in the absence and presence of Mg^2+^ are shown in Figures 4 and 5, respectively (*note*: in these experiments, the separation between the free dT*_n_* oligomers and the various SsbB·dT*_n_* complexes is less than that observed with the SsbA protein, owing to the smaller molecular size of the SsbB protein and the increased electrophoretic mobility of the SsbB·dT*_n_* complexes).

#### Absence of Mg^2+^

When increasing concentrations of SsbB were added to dT_50_ in the absence of Mg^2+^, a single complex with a gel mobility lower than that of the unbound dT_50_ was formed (B_1_ complex). All of the dT_50_ was converted to this complex at an SsbB concentration that corresponded to approximately one SsbB tetramer per dT_50_ molecule, and there was no indication of the formation of a second complex at higher SsbB concentrations (Figure 4 and additional data not shown). A similar pattern of binding was obtained with the longer oligomers, dT_65_ (gel not shown) and dT_75_ (Figure 4). These results indicated that only a single SsbB was able to bind to these oligomers under these conditions.

When the oligomer length was increased to dT_85_, a B_1_ complex was formed at lower SsbB concentrations in a manner similar to that observed with the shorter oligomers. As the concentration of SsbB was increased further, however, the B_1_ complex disappeared and a new complex with an even lower gel mobility was formed (B_2_ complex). This result indicated that a second SsbB was able to bind to dT_85_ under these conditions (Figure 4). A similar pattern of binding was observed with the longer oligomers, dT_90_, dT_100_, and dT_130_, indicating that two SsbBs were able to bind to each of these oligomers as well. There was no indication of the formation of a third complex with these oligomers, however, even at the highest concentrations of SsbB that were examined (Figure 4).

The results in Figure 4 indicated that the shortest dT*_n_* oligomer in the set that was able to bind two SsbBs in the absence of Mg^2+^ was dT_85_, and that only two SsbBs were able to bind even when the oligomer length was increased to dT_130_. These results are summarized in Table 1.

#### Presence of Mg^2+^

When increasing concentrations of SsbB were added to dT_50_ in the presence of Mg^2+^, a single complex with a gel mobility lower than that of the unbound dT_50_ was formed (B_1_ complex), with no indication of the formation of a second complex at higher SsbB concentrations (Figure 5). A similar pattern of binding was obtained with the longer oligomers, dT_65_ (gel not shown) and dT_75_ (Figure 5). These results were similar to those that were obtained in the absence of Mg^2+^ and indicated that only a single SsbB was able to bind to these oligomers under these conditions. B_1_ complexes were also formed when increasing concentrations of SsbB were added to the longer oligomers, dT_85_ and dT_90_, but in contrast to the results that were obtained in the absence of Mg^2+^, B_2_ complexes were not detected with these oligomers (Figure 5).

When the oligomer length was increased to dT_100_, a B_1_ complex was formed at lower SsbB concentrations in a manner similar to that observed with the shorter oligomers. When the concentration of SsbB was increased further, however, the B_1_ complex disappeared and a new complex of even lower gel mobility was formed (B_2_ complex). This result indicated that a second SsbB was able to bind to dT_100_ under these conditions (Figure 5). A similar pattern of binding was obtained with dT_130_, indicating that two SsbBs were able to bind to this oligomer as well. There was no indication of the formation of a third complex with these oligomers, however, even at the highest concentrations of SsbB that were examined (Figure 5).

The results in Figure 5 indicated that the shortest dT*_n_* oligomer in the set that was able to bind two SsbBs in the presence of Mg^2+^ was dT_100_, and that only two SsbBs were able to bind even when the oligomer length was increased to dT_130_. These results are summarized in Table 1.

### Co-assembly of SsbA and SsbB proteins

The ability of the SsbA and SsbB proteins to co-assemble on dT*_n_* oligomers was also investigated. For these experiments, dT*_n_* oligomers were selected that were long enough to accommodate the binding of either two SsbAs or two SsbBs, under various solution conditions (see Table 1).

#### Absence of Mg^2^

**Figure 6 pone-0024305-g006:**
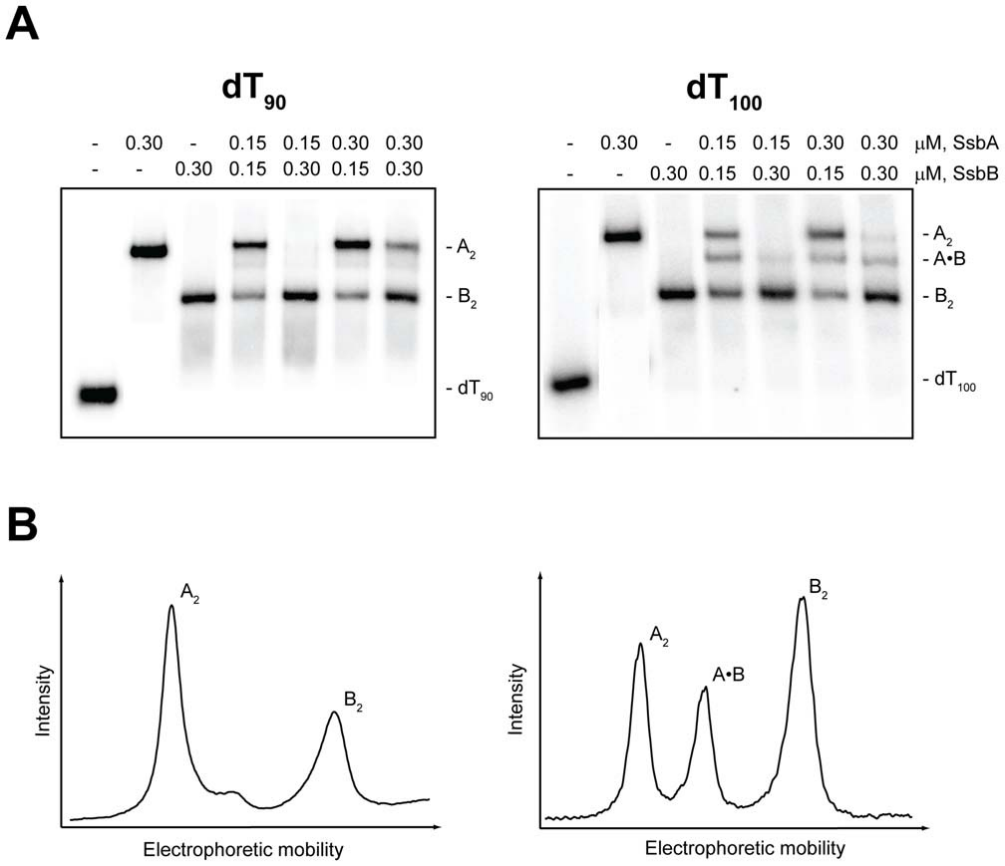
Binding of SsbA and SsbB proteins to dT_90_ and dT_100_ in the absence of Mg^2+^. The reaction solutions contained 25 mM Tris acetate (pH 7.5), 5 µM dT_90_ (*left*) or dT_100_ (*right*) (total nucleotide concentration), and the indicated concentrations of SsbA and SsbB protein (tetramer concentrations). *A,* The reactions were analyzed by polyacrylamide gel electrophoresis using a gel running buffer consisting of Tris acetate (pH 7.5). The bands corresponding to the unbound dT*_n_* oligomers, and the A_2_, B_2_, and A·B complexes, were visualized by phosphorimaging. *B,* The lanes for the reactions that contained 0.15 µM SsbA, 0.15 µM SsbB, and either dT_90_ (*left*) or dT_100_ (*right*) were scanned to show the relative intensities of the bands for the indicated complexes.

The first set of co-assembly experiments in the absence of Mg^2+^ was carried out with dT_90_ (Figure 6). When an excess concentration of SsbA alone was added to dT_90_, an A_2_ complex with two SsbAs bound to the dT_90_ was formed. Similarly, when an excess concentration of SsbB alone was added to dT_90_, a B_2_ complex with two SsbBs bound to the dT_90_ was formed. When SsbA and SsbB were added together to dT_90_, however, the A_2_ and B_2_ complexes were again formed, but little if any mixed complexes with one SsbA and one SsbB bound to the same dT_90_ molecule were detected (as judged by the absence of a new band with a mobility intermediate between that of the A_2_ and B_2_ complexes). These results indicated that although either two SsbAs or two SsbBs could bind to dT_90_ in the absence of Mg^2+^, the binding of one SsbA and one SsbB to dT_90_ was unfavorable under these conditions.

A second set of co-assembly experiments was carried out in the absence of Mg^2+^ with the longer oligomer, dT_100_ (Figure 6). When an excess concentration of either SsbA or SsbB alone was added to dT_100_, the corresponding A_2_ or B_2_ complexes were formed, as with dT_90_. In contrast to the results that were obtained with dT_90_, however, the A_2_ and B_2_ complexes, and a third complex with an intermediate mobility were formed when SsbA and SsbB were added together to dT_100_. The intermediate band was excised from the gel, analyzed by SDS-polyacrylamide gel electrophoresis, and found to contain approximately equal amounts of SsbA and SsbB protein (gel not shown). These results indicated that the intermediate band corresponded to a mixed complex in which one SsbA and one SsbB were bound to the dT_100_ (A·B complex).

The results in Figure 6 indicated that although the simultaneous binding of SsbA and SsbB to dT_90_ was unfavorable in the absence of Mg^2+^, SsbA and SsbB were able to bind together on dT_100_ under these conditions.

#### Presence of Mg^2+^

**Figure 7 pone-0024305-g007:**
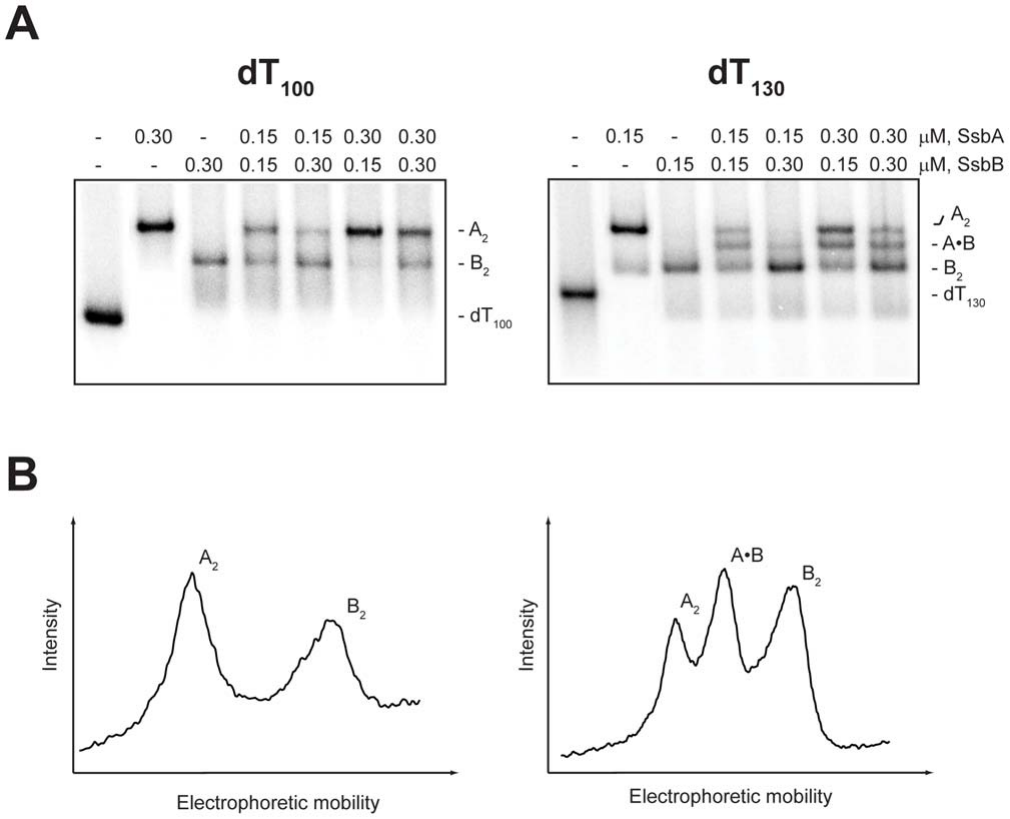
Binding of SsbA and SsbB proteins to dT_100_ and dT_130_ in the presence of Mg^2+^. The reaction solutions contained 25 mM Tris acetate (pH 7.5), 10 mM magnesium acetate, 5 µM dT_100_ (*left*) or dT_130_ (*right*) (total nucleotide concentration), and the indicated concentrations of SsbA and SsbB protein (tetramer concentrations). *A,* The reactions were analyzed by polyacrylamide gel electrophoresis using a gel running buffer consisting of Tris acetate (pH 7.5) and 10 mM magnesium acetate. The bands corresponding to the unbound dT*_n_* oligomers, and the A_2_, B_2_, and A·B complexes, were visualized by phosphorimaging. *B,* The lanes for the reactions that contained 0.15 µM SsbA, 0.15 µM SsbB, and either dT_100_ (*left*) or dT_130_ (*right*) were scanned to show the relative intensities of the bands for the indicated complexes.

The first set of co-assembly experiments in the presence Mg^2+^ (10 mM) was carried out with dT_100_ (Figure 7). When an excess concentration of either SsbA or SsbB alone was added to dT_100_, the corresponding A_2_ and B_2_ complexes were formed as expected. When SsbA and SsbB were added together to dT_100_, however, the A_2_ and B_2_ complexes were again formed, but no mixed complexes with one SsbA and one SsbB bound to the same dT_100_ molecule were detected (as judged by the absence of a new band with a mobility intermediate between that of the A_2_ and B_2_ complexes). These results indicated that although two SsbAs or two SsbBs could bind to dT_100_ in the presence of Mg^2+^, the binding of one SsbA and one SsbB to dT_100_ was unfavorable under these conditions.

A second set of co-assembly experiments was carried out in the presence of Mg^2+^ with the longer oligomer, dT_130_ (Figure 7). When an excess concentration of either SsbA or SsbB alone was added to dT_130_, the corresponding A_2_ or B_2_ complexes were formed, as with dT_100_. In contrast to the results that were obtained with dT_100_, however, the A_2_ and B_2_ complexes, and a third complex with an intermediate mobility were formed when SsbA and SsbB were added together to dT_100_. The appearance of the intermediate band was consistent with the formation of a mixed complex in which one SsbA and one SsbB were bound to the dT_130_ (A·B complex).

The results in Figure 7 indicated that although the simultaneous binding of SsbA and SsbB to dT_100_ was unfavorable in the presence of Mg^2+^, SsbA and SsbB were able to bind together on dT_130_ under these conditions.

### Co-assembly of SsbA protein with SsbA/B and SsbB^RYTP^ proteins

Additional co-assembly experiments were carried out with the SsbA protein and two SSB variants: the SsbA/B protein and the SsbB^RYTP^ protein. The SsbA/B protein is a chimeric protein in which the C-terminal domain of the SsbA protein (amino acids 106–156) has been replaced with the C-terminal domain from the SsbB protein (amino acids 105–131) [2]. The SsbB^RYTP^ protein is a modified SsbB protein in which a four-amino acid sequence from the N-terminal domain of the SsbB protein (^18^HKTN^21^) has been replaced with the corresponding sequence from the SsbA protein (^18^RYTP^21^) (Figure 1, Material and Methods). The SsbA/B protein (15,039 Da per monomer) and the SsbB^RYTP^ protein (14,963 Da per monomer) are similar in size to the SsbB protein (14,926 Da per monomer), and form complexes on dT_90_ that are clearly resolvable by gel electrophoresis from the complexes formed by the SsbA protein (17,350 Da per monomer). Experiments analogous to those carried out for the SsbA and SsbB proteins indicated that either two SsbA/Bs ((A/B)_2_ complex) or two SsbB^RYTP^s (B^RYTP^_2_ complex) were able to bind to dT_90_ in the absence of Mg^2+^.

#### SsbA/B protein

**Figure 8 pone-0024305-g008:**
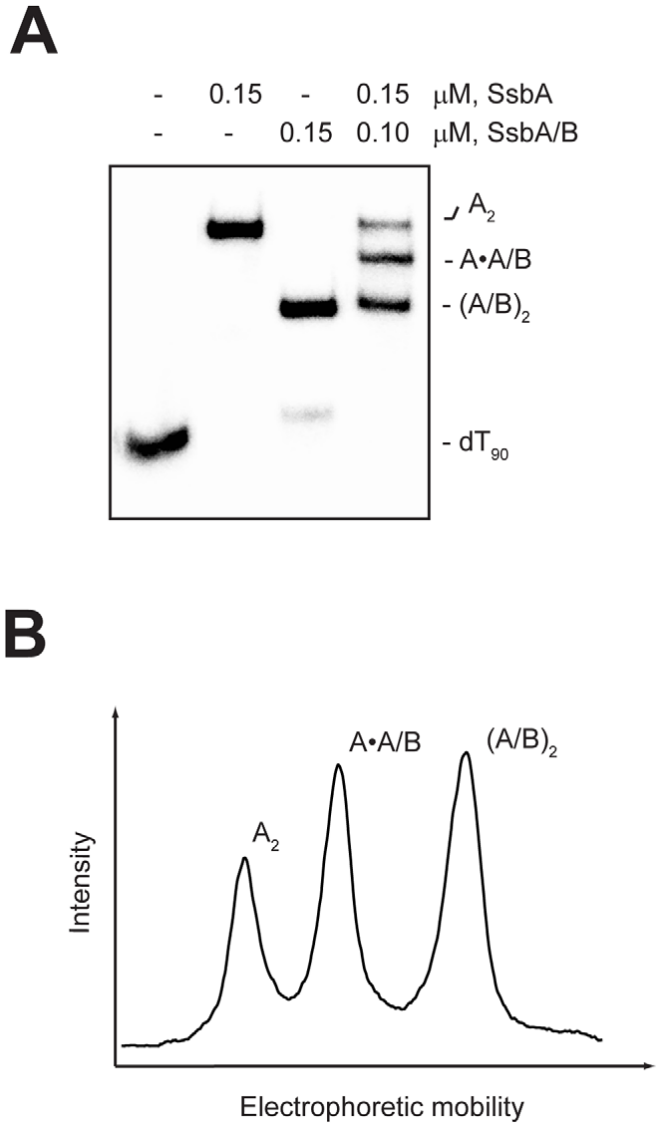
Binding of SsbA and SsbA/B proteins to dT_90_. The reaction solutions contained 25 mM Tris acetate (pH 7.5), 5 µM dT_90_ (total nucleotide concentration), and the indicated concentrations of SsbA and SsbA/B protein (tetramer concentrations). *A,* The reactions were analyzed by polyacrylamide gel electrophoresis using a gel running buffer consisting of Tris acetate (pH 7.5). The bands corresponding to the unbound dT_90_ oligomer, and the A_2_, (A/B)_2_, and A·A/B complexes, were visualized by phosphorimaging. *B,* The lane for the reaction that contained 0.15 µM SsbA and 0.10 µM SsbA/B was scanned to show the relative intensities of the bands for the indicated complexes.

The initial set of SsbA and SsbA/B co-assembly experiments was carried out with dT_90_ in the absence of Mg^2+^ (Figure 8). When an excess concentration of either SsbA or SsbA/B alone was added to dT_90_, the corresponding A_2_ and (A/B)_2_ complexes were formed, as expected. When SsbA and SsbA/B were added together to dT_90_, however, the A_2_ and (A/B)_2_ complexes, and a third complex with an intermediate mobility, were formed. The appearance of the intermediate band was consistent with the formation of a mixed complex in which one SsbA and one SsbA/B were bound to the dT_90_ (A·A/B complex).

These results indicated that although the simultaneous binding of SsbA and SsbB was unfavorable (Figure 6), SsbA was able to form a mixed complex with SsbA/B on dT_90_ in the absence of Mg^2+^ (Figure 8). Additional experiments indicated that SsbA/B also differed from SsbB it that it was able to form a mixed complex with SsbA on dT_100_ in the presence of Mg^2+^ (gel not shown).

#### SsbB^RYTP^ protein

**Figure 9:**
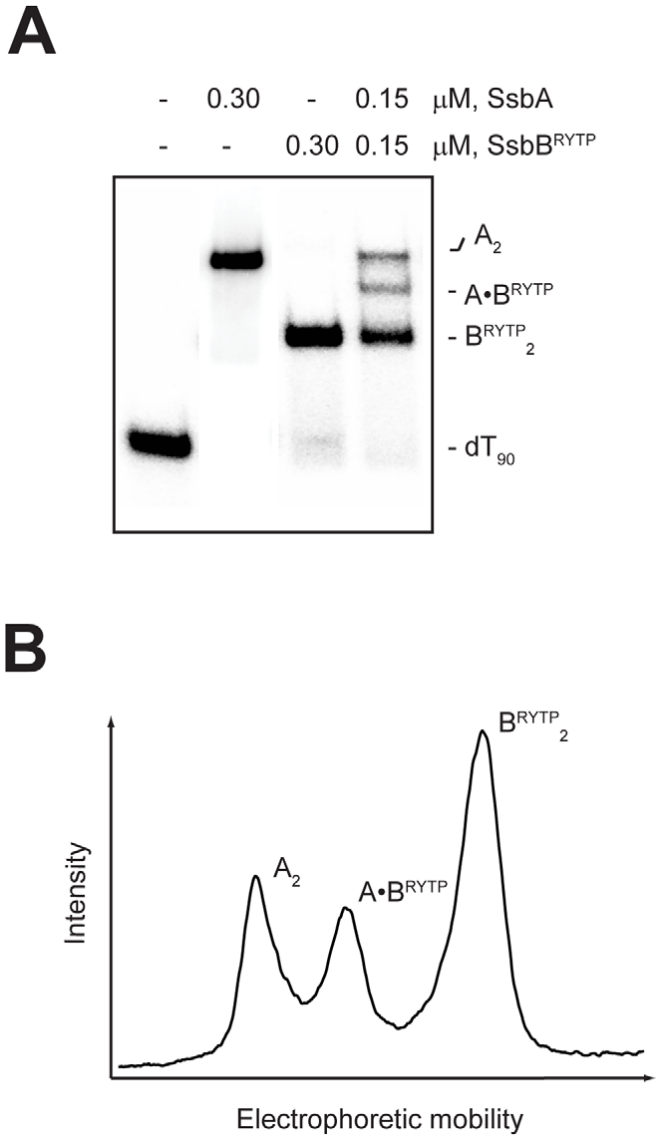
Binding of SsbA and SsbB^RYTP^ proteins to dT_90_. The reaction solutions contained 25 mM Tris acetate (pH 7.5), 5 µM dT_90_ (total nucleotide concentration), and the indicated concentrations of SsbA and SsbB^RYTP^ protein (tetramer concentrations). *A,* The reactions were analyzed by polyacrylamide gel electrophoresis using a gel running buffer consisting of Tris acetate (pH 7.5). The bands corresponding to the unbound dT_90_ oligomer, and the A_2_, B^RYTP^_2_, and A•B^RYTP^ complexes, were visualized by phosphorimaging. *B,* The lane for the reaction that contained 0.15 µM SsbA and 0.15 µM SsbB^RYTP^ was scanned to show the relative intensities of the bands for the indicated complexes.

The initial set of SsbA and SsbB^RYTP^ co-assembly experiments was also carried out with dT_90_ in the absence of Mg^2+^ (Figure 9). When an excess concentration of either SsbA or SsbB^RYTP^ alone was added to dT_90_, the corresponding A_2_ and B^RYTP^_2_ complexes were formed, as expected. When SsbA and SsbB^RYTP^ were added together to dT_90_, however, the A_2_ and B^RYTP^_2_ complexes, and a third complex with an intermediate mobility were formed. The appearance of the intermediate band was consistent with the formation of a mixed complex in which one SsbA and one SsbB^RYTP^ were bound to the dT_90_ (A·B^RYTP^ complex).

These results indicated that although the simultaneous binding of SsbA and SsbB was unfavorable (Figure 6), SsbA was able to form a mixed complex with SsbB^RYTP^ on dT_90_ in the absence of Mg^2+^ (Figure 9). Additional experiments, however, indicated that SsbB^RYTP^, like SsbB, was unable to form a mixed complex with SsbA on dT_100_ in the presence of Mg^2+^ (gel not shown).

## Discussion

The results presented here indicate that the shortest dT*_n_* oligomer that is able to accommodate the binding of two SsbAs or two SsbBs is strictly defined, and depends on whether Mg^2+^ is included in the reaction solution (Table 1). This finding suggests that the minimal oligomer length may be determined by the preferred binding modes and potential binding capacities of the individual SSB proteins under the particular solution conditions.

### SsbA protein assembly

The shortest dT*_n_* oligomer that was able to bind two SsbAs in the absence of Mg^2+^ was dT_75_ (Table 1). Assuming that SsbA interacts with dT*_n_* oligomers as a tetramer in an SSB_35_-like mode in the absence of Mg^2+^ (see Introduction), two SsbAs may assemble on dT_75_ under these conditions in a manner in which each of the SsbAs interacts with a ∼35-nucleotide segment of the oligomer. The observation that only two SsbAs were able to bind even when the oligomer length was increased to dT_100_, whereas three SsbAs were able to bind to dT_130_ indicates that at least a ∼35 nucleotide segment of dT*_n_* was required for the stable binding of each SsbA (Table 1). In all cases, as the concentration of SsbA was increased, the dT*_n_* complex with a lesser number of SsbAs bound was replaced completely by the complex with the greater number of SsbAs bound. These results indicate that under SSB_35_-like binding mode conditions, individual SsbAs can organize themselves so as to maximize the number of SsbAs bound to a dT*_n_* oligomer while satisfying the ∼35-nucleotide binding requirement of each bound SsbA.

**Figure 10:**
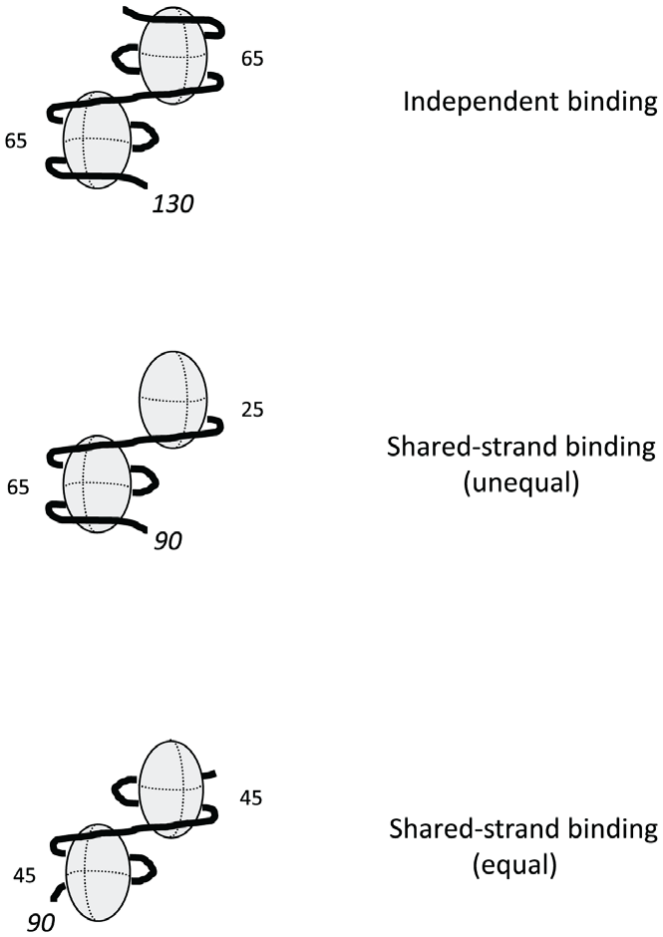
Independent and shared-strand DNA binding complexes. The first complex represents an *independent binding* complex in which two SSB tetramers are each bound to a 65-nucleotide segment of a dT_130_ molecule. The second complex represents a *shared-strand binding* complex in which one SSB tetramer is bound to a 65-nucleotide segment and the second SSB tetramer is bound to the remaining 25-nucleotide segment of a dT_90_ molecule (*unequal sharing*). The third complex represents a *shared-strand binding* complex in which two SSB tetramers are each bound to a 45-nucleotide segment of a dT_90_ molecule (*equal sharing*). The paths shown for the dT*_n_* strands in these complexes are speculative and are based on a structural model of the SsbEc protein bound to single-stranded DNA in the SSB_65_ binding mode [7].

A longer dT*_n_* oligomer was required for the binding of two SsbAs when Mg^2+^ was included in the reaction solution (Table 1). Assuming that SsbA interacts with dT*_n_* oligomers as a tetramer in an SSB_65_-like binding mode in the presence of Mg^2+^ (see Introduction), it is likely that the increased length requirement is due to the higher binding capacity of SsbA under these conditions. The complexes containing two SsbAs that were observed with dT_130_ are consistent with an SSB_65_-like mode of binding in that dT_130_ is long enough to satisfy the full capacity of ∼65 nucleotides expected for each of the bound SsbAs (∼130 nucleotides total) (Figure 10). However, stable complexes containing two SsbAs could also be formed on oligomers as short as dT_90_ under these conditions (Table 1). With these shorter oligomers, one or both of the SsbAs were presumably bound using only a portion of their potential binding capacity. These results suggest that under SSB_65_-like binding mode conditions, two SsbAs are able to assemble onto shorter segments of single-stranded DNA via a coordinated sharing of the DNA strand between the bound proteins.

Various arrangements can be envisioned for the DNA strand in a “shared-strand binding” mechanism (Figure 10). With dT_90_ for example, one SsbA could be bound to a ∼65-nucleotide segment, with the second SsbA bound to the remaining ∼25-nucleotide segment of the oligomer. Alternatively, the dT_90_ may be more equally shared between the two SsbAs, with each binding to a ∼45-nucleotide segment of the oligomer. These possibilities are not necessarily mutually exclusive and a combination of different binding arrangements may also occur. In any case, the observation that complexes with two SsbAs were not formed on dT*_n_* oligomers shorter than dT_90_ when Mg^2+^ was included in the reaction solution, but could be formed on oligomers as short as dT_75_ in absence of Mg^2+^ (Table 1) suggests that the shared-strand arrangement that is adopted when two SsbAs bind in the SSB_65_-like mode may be different from the binding arrangement that is used when two SsbAs bind in the SSB_35_-like mode.

### SsbB protein assembly

Longer dT*_n_* oligomers were required for the binding of two SsbBs than were needed for two SsbAs, both in the absence and presence of Mg^2+^ (Table 1). Assuming that SsbB interacts with dT*_n_* oligomers as a tetramer in an SSB_65_-like mode in the absence of Mg^2+^ and in an enhanced SSB_65_-like mode in the presence of Mg^2+^ (see Introduction), the longer length requirement may reflect the higher binding capacity of SsbB, relative to that of SsbA, under these reaction conditions. The complexes containing two SsbBs that were observed with dT_130_ in both the absence and presence of Mg^2+^ are consistent with either the SSB_65_-like mode or the enhanced SSB_65_-like mode of binding in that dT_130_ is long enough to satisfy the full ∼65 nucleotide binding capacity expected for each of the bound SsbBs in either case (∼130 nucleotides total) (Figure 10). However, stable complexes containing two SsbBs could also be formed on oligomers as short as dT_85_ (in the absence of Mg^2+^) or dT_100_ (in the presence of Mg^2+^). These results suggest that two SsbBs can bind to the shorter oligomers using a shared-strand binding mechanism analogous to that proposed for SsbA in the presence of Mg^2+^ (SSB_65_-like binding mode conditions) (Figure 10). In the case of SsbB, the observation that dT_85_ was able to accommodate the binding of two SsbBs in absence of Mg^2+^, whereas at least dT_100_ was required for the binding of two SsbBs in the presence of Mg^2+^ may reflect a difference in the arrangement of the shared strand between the two SsbBs under normal SSB_65_-like mode versus enhanced SSB_65_-like mode binding conditions.

### SsbA and SsbB protein co-assembly

The shortest dT*_n_* oligomer that was able to accommodate the simultaneous binding of one SsbA and one SsbB was also strictly defined and depended on the solution conditions.

In the absence of Mg^2+^, complexes containing either two SsbAs or two SsbBs were readily formed on dT_90_. However, little or no mixed complexes with one SsbA and one SsbB were detected when both proteins were added together to dT_90_ (Figure 6). If the binding capacity of SsbA under these conditions is assumed to be ∼35 nucleotides (SSB_35_-like mode) and the binding capacity of SsbB is assumed to be ∼65 nucleotides (SSB_65_-like mode), a dT_90_ molecule would not be long enough to satisfy the full binding capacities of one SsbA and one SsbB (∼100 nucleotides total). Therefore, the simultaneous binding of SsbA and SsbB to dT_90_ would presumably require one or both of these proteins to bind using only a portion of their potential binding capacity. The absence of mixed complex formation on dT_90_ thus suggests that SsbA and SsbB are not able to engage in shared-strand binding in the absence of Mg^2+^.

The apparent incompatibility in shared-strand binding does not appear to preclude SsbA and SsbB from binding independently on longer dT*_n_* oligomers where strand sharing would not be required. Although they were unable to co-assemble on dT_90_, SsbA and SsbB were able to form a mixed complex on dT_100_ in the absence of Mg^2+^ (Figure 6). In this case, a dT_100_ molecule could potentially provide a ∼35-nucleotide segment for the SsbA and a ∼65-nucleotide segment for the SsbB, and thereby satisfy the full binding capacities of both proteins under the reaction conditions.

DNA binding compatibility also appears to govern the co-assembly of SsbA and SsbB on dT*_n_* oligomers when Mg^2+^ is included in the reaction solution. Although complexes with two SsbAs or two SsbBs were readily formed on either dT_100_ or dT_130_ in the presence of Mg^2+^, mixed complexes with one SsbA and one SsbB were detected only with dT_130_ (Figure 7). If the binding capacity of SsbA under these conditions is assumed to be ∼65 nucleotides (SSB_65_-like mode) and the binding capacity of SsbB is also assumed to be ∼65 nucleotides (enhanced SSB_65_-like mode), a dT_130_ molecule would be able to satisfy the full binding capacities of one SsbA and one SsbB (∼130 nucleotides total), whereas a dT_100_ molecule would only be able to partially satisfy the binding capacities of the two proteins. Thus, the formation of mixed complexes on dT_130_, but not on dT_100_, indicates that SsbA and SsbB are able to bind independently, but are not able to engage in shared-strand binding, in the presence of Mg^2+^.

### SsbA/B and SsbB^RYTP^ proteins

Although SsbA and SsbB appeared to be incompatible for shared-strand binding, SsbA was able to form mixed complexes under some shared-strand binding conditions with two SSB protein variants: the SsbA/B protein and the SsbB^RYTP^ protein.

The SsbA/B protein, in which the C-terminal domain of the SsbA protein has been replaced with the C-terminal domain from the SsbB protein, was prepared previously to assess the contribution of the C-terminal domains to the DNA binding properties of the SsbA and SsbB proteins [2]. The DNA binding mode preferences of the SsbA/B protein were found to be similar to those of the SsbA protein, suggesting that the primary structural determinants of DNA binding may be contained within the N-terminal domains of the various SSB proteins [2]. Moreover, the SsbA/B protein was able to form a mixed complex with SsbA on dT_90_ in the absence of Mg^2+^ and on dT_100_ in the presence of Mg^2+^ (Figure 8, and gel not shown). These findings suggest that the inability of SsbA to engage in shared-strand binding with SsbB may not be due to the dissimilar C-terminal domain of the SsbB protein, inasmuch as the C-terminal domain of the SsbA/B protein is identical to that of the SsbB protein. It is possible, however, that the C-terminal domain functions differently in the SsbB protein than in the chimeric SsbA/B protein, and contributes to the incompatibility of SsbA and SsbB in shared-strand binding.

The SsbB^RYTP^ protein, in which the ^18^HKTN^21^ sequence of the SsbB protein has been replaced with the corresponding ^18^RYTP^21^ sequence from the SsbA protein, was also prepared in an effort to determine the structural basis for the differential DNA binding properties of the SsbA and SsbB proteins. The DNA binding properties of the SsbB^RYTP^ protein were found to be similar to those of the SsbB protein, indicating that the ^18^HKTP^21^ sequence may not be responsible for the distinctive DNA binding mode preferences of the SsbB protein (see Experimental Procedures). The SsbB^RYTP^ protein differed from the SsbB protein, however, in that it was able to form a mixed complex with SsbA on dT_90_ in the absence of Mg^2+^ (Figure 9). These results suggest that the shared-strand binding incompatibility that was observed with the SsbA and SsbB proteins in absence of Mg^2+^ was not due to the difference in their preferred DNA binding modes, but may be attributable to the divergent ^18^RYTP^21^ and ^18^HKTN^21^ sequences of these proteins. The observation that SsbB^RYTP^ was unable to form a mixed complex with SsbA on dT_100_ in the presence of Mg^2+^, however, indicates that this sequence difference is not sufficient to account for the shared-strand binding incompatibility that was observed with SsbA and SsbB in the presence of Mg^2+^. Thus, the introduction of the ^18^RYTP^21^ sequence into the SsbB protein has the effect of uncoupling the Mg^2+^-independent shared-strand binding incompatibility from the Mg^2+^-dependent incompatibility.

**Figure 11:**
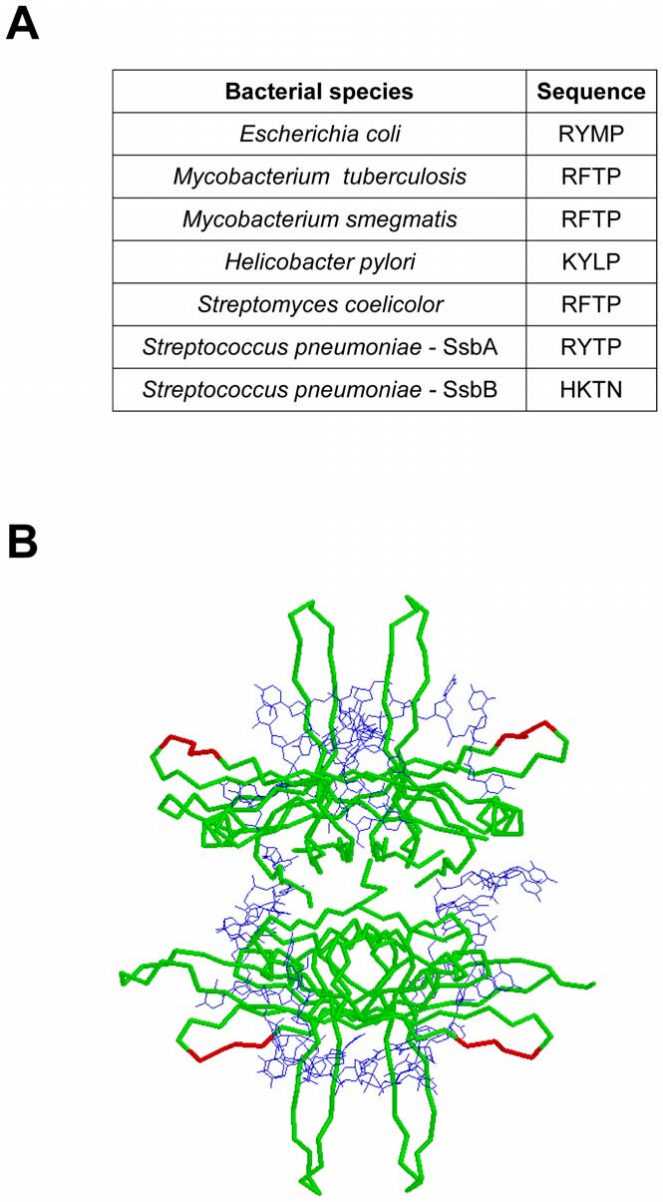
Loop 1 region of bacterial SSB proteins. *A,* Comparison of sequences from the Loop 1 region of the SSB proteins from *Escherichia coli* (PDB code 1eyg), *Mycobacterium tuberculosis* (PDB code 1ue1), *Mycobacterium smegmatis* (PDB code 1x3e), *Helicobacter pylori* (PDB code 2vw9), and *Streptomyces coelicolor* (PDB code 3eiv) with the corresponding sequences from the *Streptococcus pneumoniae* SsbA and SsbB proteins. *B,* Structural model for an SsbEc tetramer (*green*)-single-stranded DNA (*blue*) complex (based on PDB code 1eyg) [7]. The RYMP sequence in the Loop 1 region of each SsbEc subunit is highlighted (*red*).

The ^18^RYTP^21^ sequence of the SsbA protein is at least partially conserved in the SsbEc protein and in a number of other homotetrameric bacterial SSB proteins whose x-ray crystal structures have been determined (Figure 11). An inspection of the various structures shows that the tertiary folds of the N-terminal domains are similar in all cases, and that the ^18^RYTP^21^ sequence of the SsbA protein, and the divergent ^18^HKTN^21^ sequence of the SsbB protein, correspond to the Loop 1 region on the outer surface of the subunits of the SSB tetramers (Figure 11) [6]–[7]. The differences in the composition of the Loop 1 region may account for the inability of the SsbA and SsbB proteins to engage in shared-strand binding in the absence of Mg^2+^. For example, the Loop 1 variations could conceivably affect the precise orientation of the DNA strand as it winds around the individual SSB tetramers, or influence the manner in which two SSB tetramers interact when bound in close proximity on a DNA strand. Other molecular determinants are apparently required, however, for shared-strand binding compatibility in the presence of Mg^2+^. A definitive determination of the molecular basis for shared-strand binding and DNA binding compatibility will require further structural analysis of the various SSB-dT*_n_* complexes. These studies are currently underway in our laboratory.

### SsbA and SsbB binding compatibility

SsbA is a constitutively-expressed protein, and presumably functions as the primary SSB protein during the routine replication and maintenance of chromosomal DNA in *S. pneumoniae* (analogous to the SsbEc protein in *E. coli*). SsbB, in contrast, is induced specifically during natural transformation, and associates transiently with a single-stranded form of the exogenous DNA before the DNA is incorporated into a homologous region of the *S. pneumoniae* chromosome (there is no analog of the SsbB protein in *E.coli*) [1], [8].

The extent to which the SsbA and SsbB proteins are functionally interchangeable in these various activities is not clear. Our results, however, indicate that the SsbA and SsbB proteins will be able to bind together on longer single-stranded DNA segments where independent binding is possible, but suggest that they may not co-assemble on shorter single-stranded DNA segments where coordinated interactions between adjacent SSBs are required. The compatibility requirement for shared-strand binding could conceivably serve as a self-recognition mechanism that regulates the manner in which SsbA and SsbB interact in *S. pneumoniae*.

## Materials and Methods

### Materials

*S. pneumoniae* SsbA protein [9], SsbB protein [10], and SsbA/B protein [2] were prepared as previously described. Gel-purified dT*_n_* oligomers were from Invitrogen. ^32^P-end-labeled dT*_n_* oligomers were prepared using [γ-^32^P]ATP (PerkinElmer) and T4 polynucleotide kinase (New England Biolabs).

### Preparation and characterization of the SsbB^RYTP^ protein

The SsbB^RYTP^ protein coding sequence, in which the nucleotide sequence of the SsbB protein corresponding to amino acids ^18^HKTN^21^ was replaced with a sequence coding for the amino acids ^18^RYTP^21^, was generated by overlap-extension PCR mutagenesis. The initial mutagenesis template was our previously described pETssbB construct, which contains the wild type SsbB sequence cloned into the *NdeI*/*HindIII* site of the pET21a expression vector (Novagen) [10]. Primer *a* (5′-CGGATAACAATTCCCCTCTAG-3′) and primer *d* (5′-TTAGCAGCCGGATCTCAGTGG-3′) flanked the *ssbB* gene, and primer *b* (5′-CTTGTCATTTGGAGTGTAACGCAATTCTGGTGTAGAC-3′) and primer *c* (5′-GAATTGCGTTACACTCCAAATGACAAGTCGGTAGC-3′) were the internal overlapping mutagenic primers (mutagenic bases are underlined). The final SsbB^RYTP^-coding PCR product was digested with *NdeI* and *HindIII* and then cloned into the *NdeI*/*HindIII* site of a pET21a expression vector to yield the construct pETssbB^RYTP^. The insert was sequenced and found to be identical to the expected SsbB^RYTP^ protein coding sequence.

The pETssbB^RYTP^ expression plasmid was introduced into *E. coli* strain Rosetta(DE3)pLysS (Novagen), and the SsbB^RYTP^ protein was purified from the resulting Rosetta(DE3)pLysS/pETssbB^RYTP^ cells using a procedure analogous to that described previously for the wild type SsbB protein [10]. The final fraction of SsbB^RYTP^ protein was greater than 95% pure as judged by SDS-polyacrylamide gel electrophoresis.

The purified SsbB^RYTP^ protein was characterized using the dT_35_ binding assay that was described previously for the SsbA and SsbB proteins [2]. The results were similar to those that were obtained with the wild type SsbB protein, and indicated that the SsbB^RYTP^ protein was able to bind two dT_35_ molecules in the absence of Mg^2+^, and two dT_35_ molecules (with positive intersubunit cooperativity) in the presence of 10 mM Mg^2+^.

### Polyacrylamide gel electrophoresis assays

The dT*_n_* binding reaction solutions (30 µl) contained 25 mM Tris acetate (pH 7.5), 5% glycerol, 1 mM dithiothreitol, and the concentrations of magnesium acetate, dT*_n_* (^32^P-end-labeled), and SSB protein given in the figure legends. The reactions solutions were incubated at 25°C for 15 min, and then 3 µl of gel loading solution (0.25% bromophenol blue, 40% sucrose) was added. An aliquot (20 µl) of the final solution was analyzed by electrophoresis on 5% native polyacrylamide gels using a buffer system consisting of 25 mM Tris acetate (pH 7.5) and the same concentration of magnesium acetate as in the reaction solutions. The bands corresponding to unbound and SSB-bound dT*_n_* oligomers were visualized using a Fuji FLA-7000 imager. The specific protein concentrations for the individual gels in Figures 2–9 were selected to illustrate the concentration-dependent formation of the various SSB-dT*_n_* complexes.
